# Supplementary figures and images for: Press Disturbance Alters Community Structure and Assembly Mechanisms of Bacterial Taxa and Functional Genes in Mesocosm-Scale Bioreactors
Source: mSystems. 2020 Aug 25;5(4):e00471-20. doi: 10.1128/mSystems.00471-20 (PMC7449608; doi:10.1128/mSystems.00471-20)

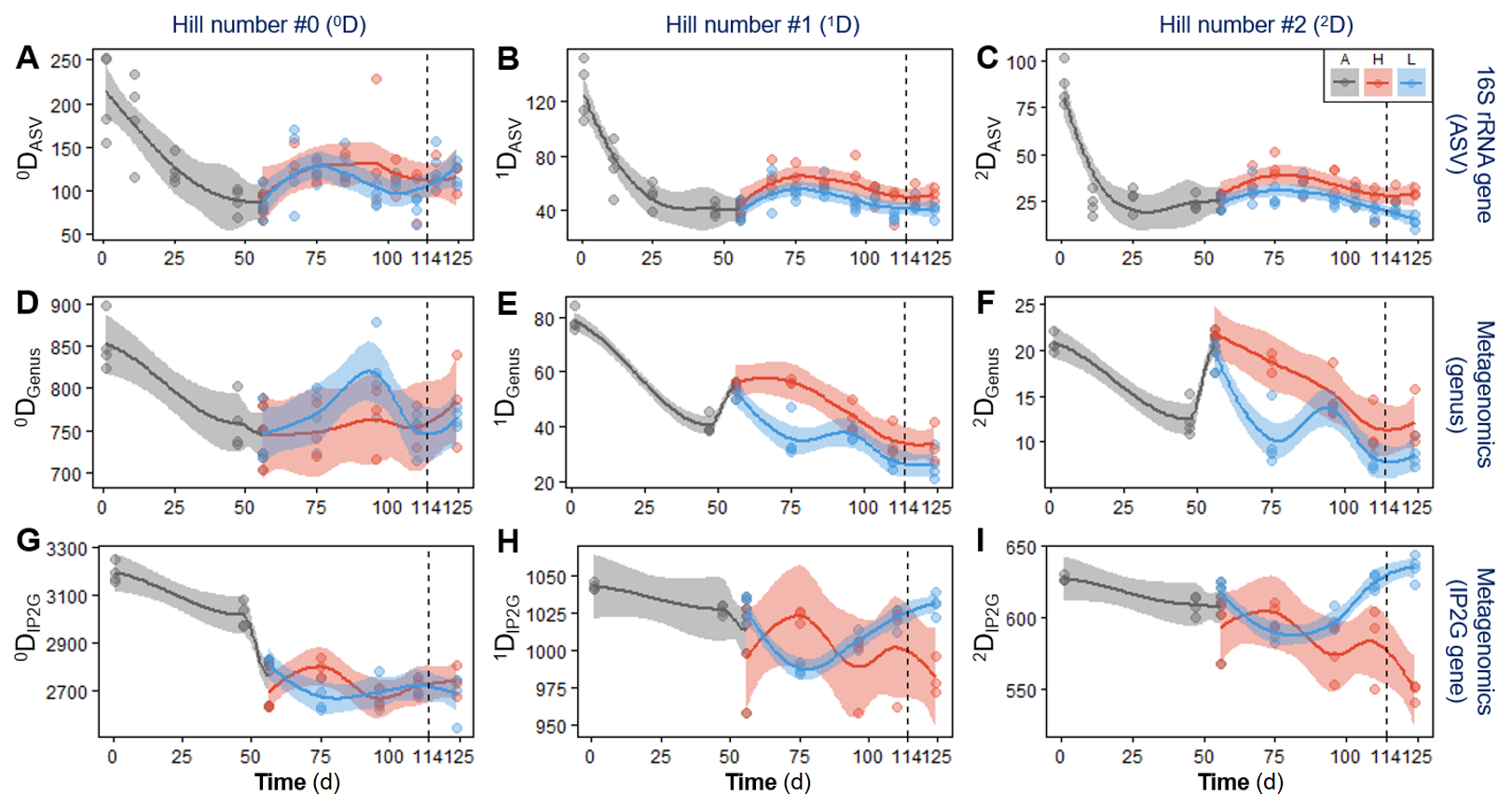

Supplement: FIG S1 [file mSystems.00471-20-sf001.tif]

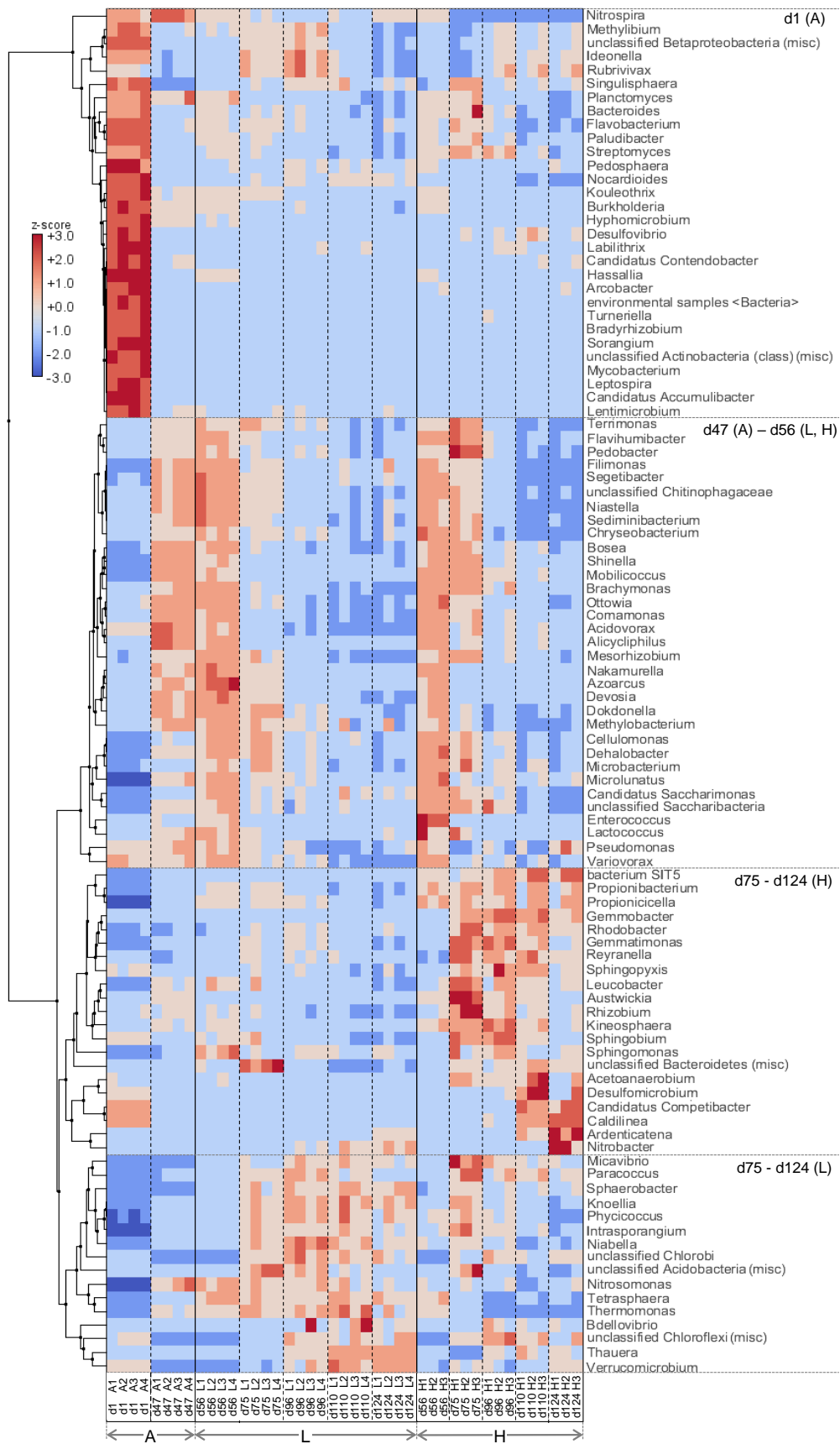

Supplement: FIG S2 [file mSystems.00471-20-sf002.pdf]

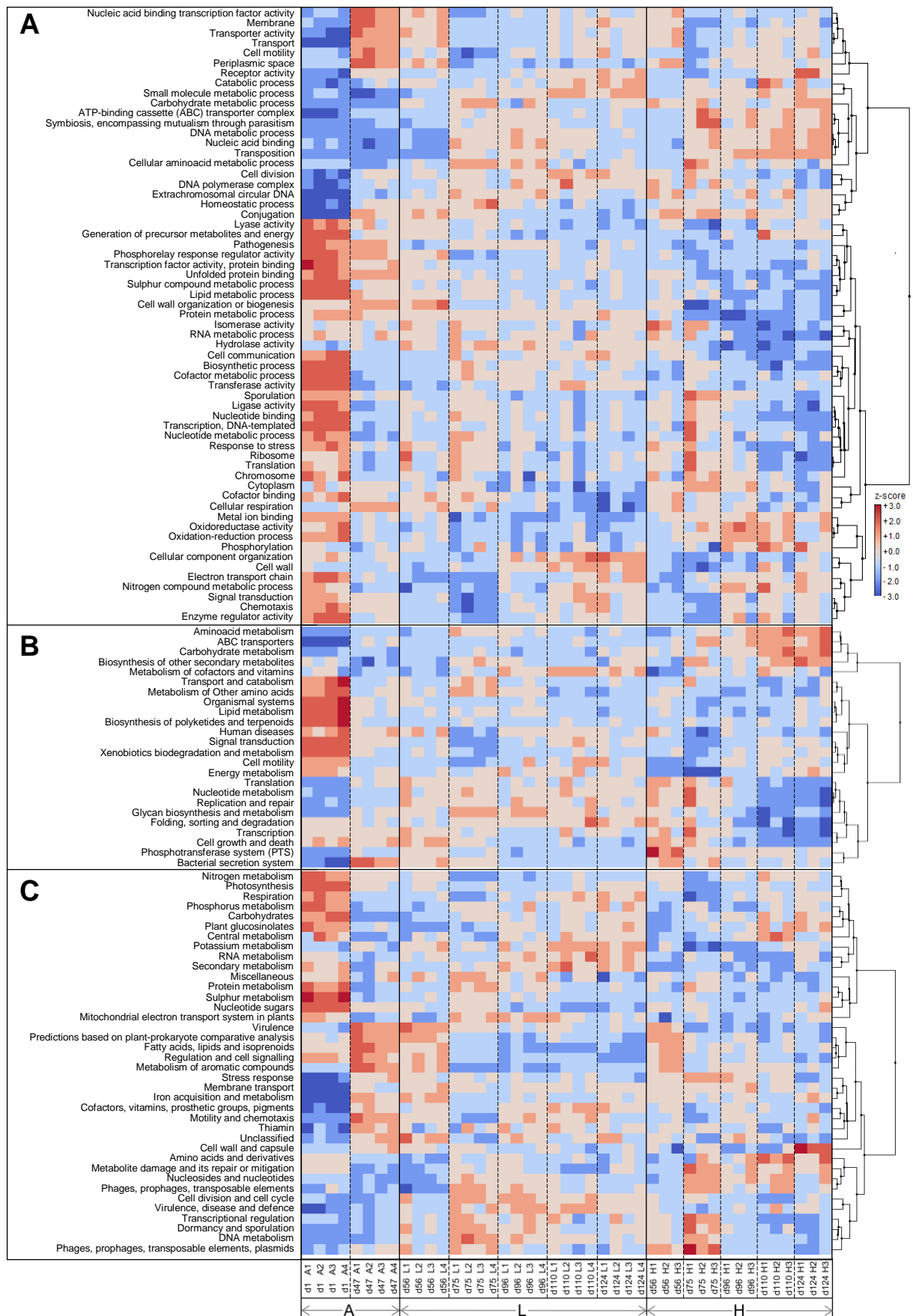

Supplement: FIG S3 [file mSystems.00471-20-sf003.pdf]

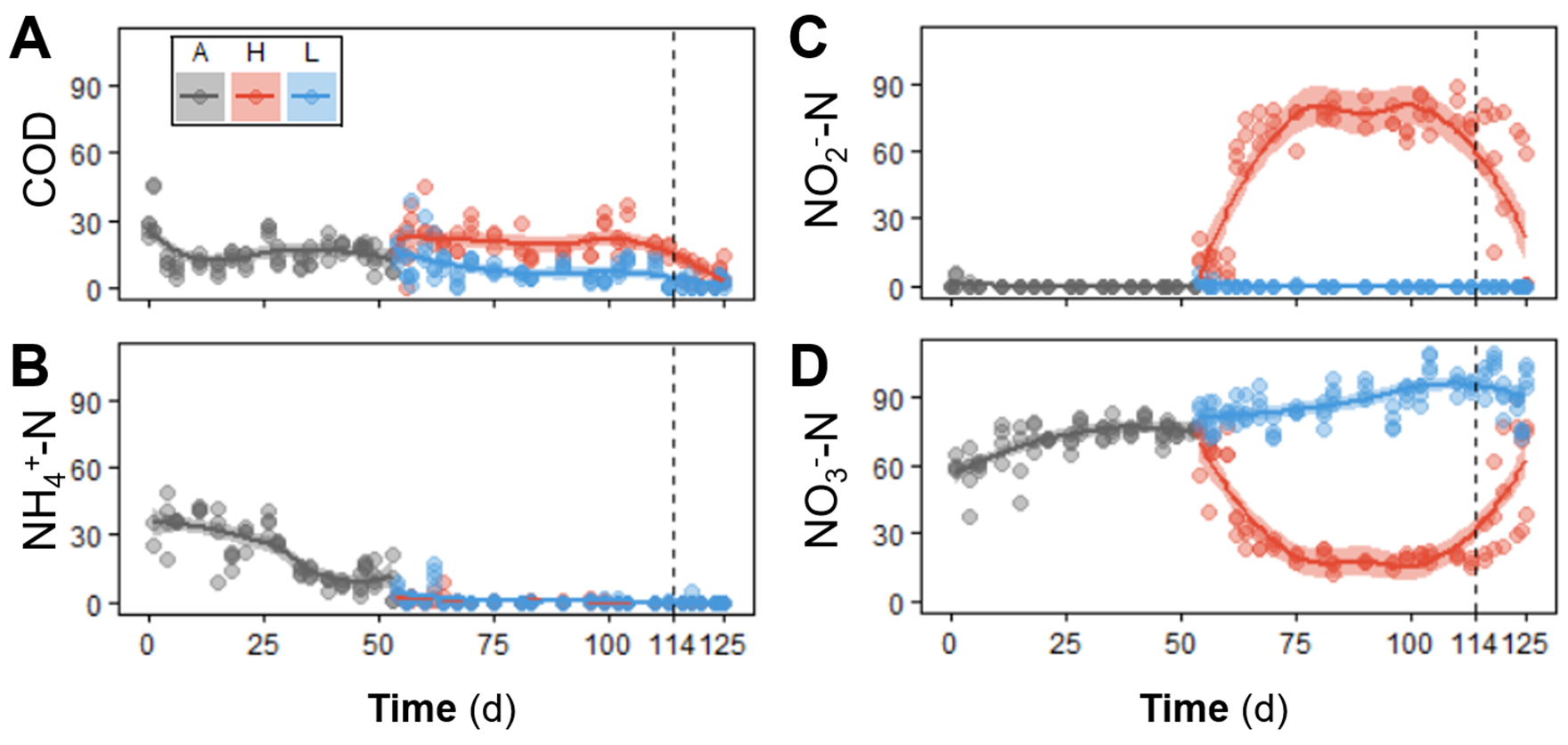

Supplement: FIG S4 [file mSystems.00471-20-sf004.tif]

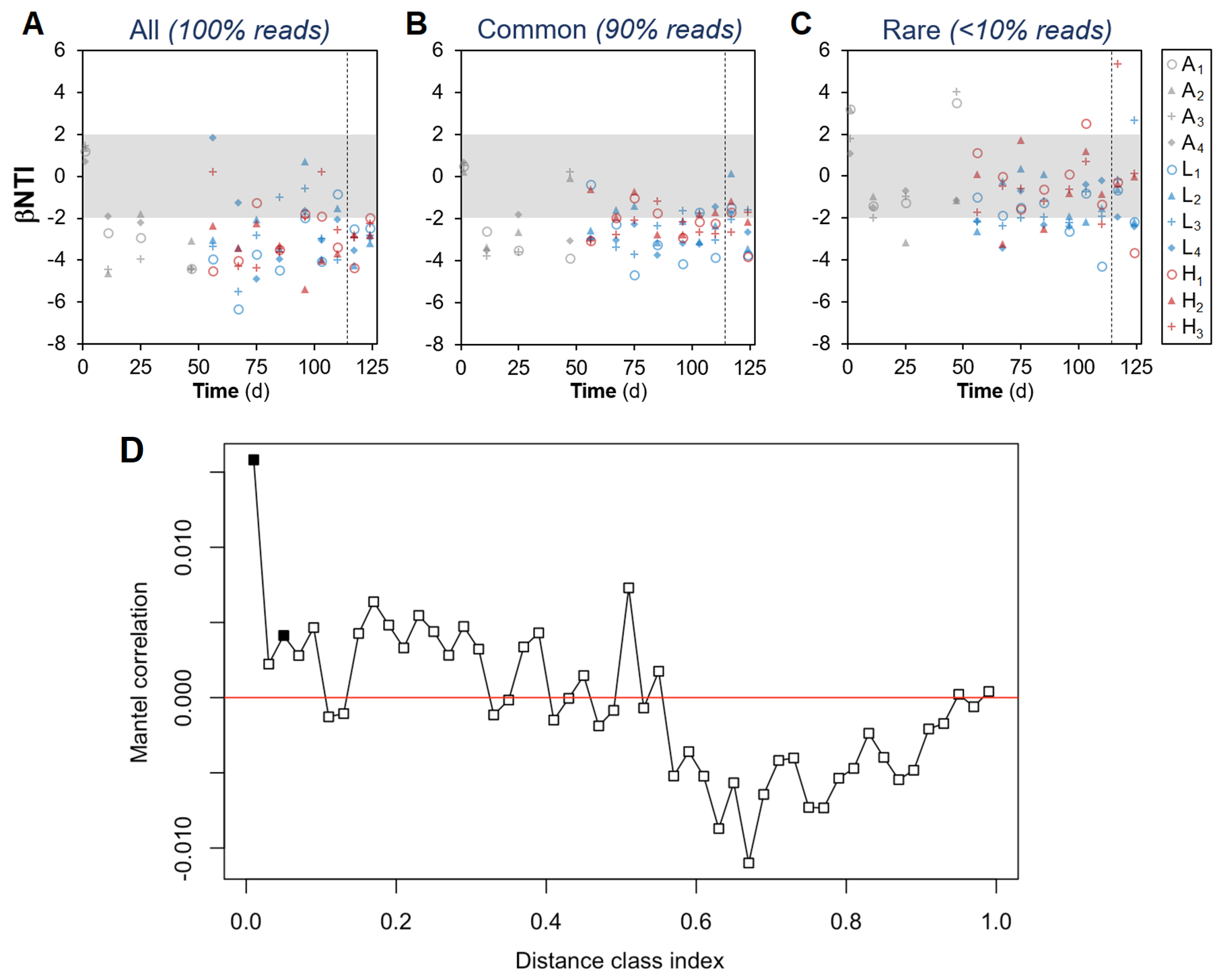

Supplement: FIG S5 [file mSystems.00471-20-sf005.tif]

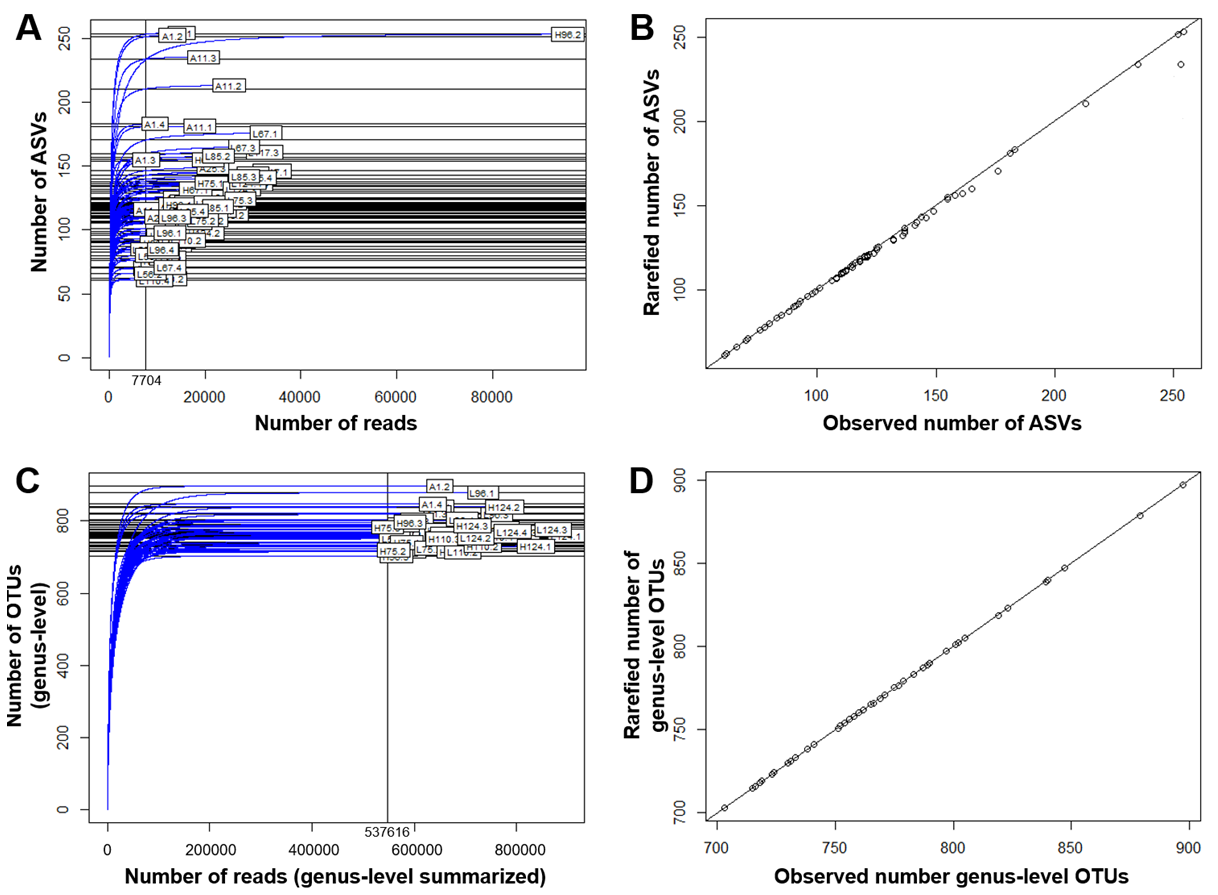

Supplement: FIG S6 [file mSystems.00471-20-sf006.tif]
