# Supplementary material for: Press Disturbance Alters Community Structure and Assembly Mechanisms of Bacterial Taxa and Functional Genes in Mesocosm-Scale Bioreactors
Source: mSystems. 2020 Aug 25;5(4):e00471-20. doi: 10.1128/mSystems.00471-20 (PMC7449608; doi:10.1128/mSystems.00471-20)
Supplement: TEXT S1 [file mSystems.00471-20-s0001.pdf]

Supplemental Text

Press disturbance alters community structure and assembly mechanisms of bacterial taxa and functional genes in mesocosm-scale bioreactors

Authors: Ezequiel Santillan[1,2], Florentin Constancias[1], and Stefan Wuertz[1,2,3]*

Affiliations:
[1]Singapore Centre for Environmental Life Sciences Engineering, Nanyang Technological University, 637551, Singapore.
[2]Department of Civil and Environmental Engineering, University of California, Davis, CA 95616, U.S.A.
[3]School of Civil and Environmental Engineering, Nanyang Technological University, 639798, Singapore.
*Correspondence to: Stefan Wuertz, swuertz@ntu.edu.sg

Stabilization during acclimation phase

Sludge acclimation was a necessary transient stage in terms of ecosystem function and community structure. This period allowed for important functions like nitrification and organic carbon removal to stabilize across reactors (Fig. S4), as previously suggested[1]. We observed that α-diversity for both bacterial genera and functional genes decreased during acclimation (Fig. 1), which is coherent with the shift in β-diversity clusters (Fig. 2A). A decrease in bacterial diversity in bioreactors is expected, due to radically changing conditions when communities are moved from a full-scale plant to a laboratory setting, and has been observed for both anaerobic[2] and aerobic[3] bioreactors.

Bacterial genus-level structure dynamics

Analysis of changes in the 100 most abundant genera via metagenomics also revealed bacterial succession in different clusters (Fig. S2). Taxa that prevailed in the sludge inoculum (d1) like *Ca.* Accumulibacter, *Desulfovibrio,* and *Mycobacterium,* decreased to very low levels of relative abundance during the remainder of the study. At the end of the acclimation phase (d47) and at the beginning of the experimental phase (d56), *Bosea*, *Acidovorax*, *Nakamurella*, *Comamonas,* and *Microlunatus* dominated. The organisms prevailing in disturbed and undisturbed reactors also varied from d75 onwards. *Tetrasphaera*, *Nitrosomonas*, and *Thauera* preferred the low organic loading conditions, whereas genera like *Gemmatimonas*, *Propionibacterium*, and *Kineosphaera* prevailed

at high organic loading. After the switch back from high to low organic loading, *Ca.* Competibacter and *Caldilinea* showed an increase in abundance to levels comparable to the ones found in the inoculum.

Richness is not recommended as α-diversity metric for microbial community studies

In Fig. 1 we chose to display only the 2nd order true α-diversity (^{2}D) because of two reasons: (i) For complex communities there is often a huge difference between the abundance of rare and abundant taxa, and overall changes in communities are driven by changes in the most abundant taxa[4], which in terms of α-diversity are best described by ^{2}D α-diversity metric[5]. (ii) The use of richness (first order α-diversity, ^{0}D) to describe microbial communities is not reliable since it is heavily constrained by the method of measurement[5], which makes the comparison of results from different sequencing techniques using this metric meaningless[6]. For example, the increase in $^{0}D_{Genus}$ diversity for low organic loading reactors during the disturbance phase (Fig. S1D) likely means that more genera increased in abundance above the limit of detection, as new organisms could not be incorporated since our bioreactors system was closed to immigration. The latter implies that these taxa were already in the reactors but at levels below the detection limit. This phenomenon could happen during any study on complex microbial communities leading to spurious patterns without ecological meaning. It is, therefore, not recommended to draw conclusions based on bacterial richness dynamics.

Supplementary References

1 Falk, M. W., Song, K. G., Matiasek, M. G. & Wuertz, S. (2009) Microbial community dynamics in replicate membrane bioreactors - Natural reproducible fluctuations. *Water Res.* **43**, 842-852.

2 Vanwonterghem, I., Jensen, P. D., Dennis, P. G., Hugenholtz, P., Rabaey, K. & Tyson, G. W. (2014) Deterministic processes guide long-term synchronised population dynamics in replicate anaerobic digesters. *ISME J.* **8**, 2015.

3 Santillan, E., Seshan, H., Constancias, F., Drautz-Moses, D. I. & Wuertz, S. (2019) Frequency of disturbance alters diversity, function, and underlying assembly mechanisms of complex bacterial communities. *NPJ Biofilms Microbiomes* **5**, 1-8.

4 van Dorst, J., Bissett, A., Palmer, A. S., Brown, M., Snape, I., Stark, J. S., Raymond, B., McKinlay, J., Ji, M. K., Winsley, T. & Ferrari, B. C. (2014) Community fingerprinting in a sequencing world. *FEMS Microbiol. Ecol.* **89**, 316-330.

5 Haegeman, B., Hamelin, J., Moriarty, J., Neal, P., Dushoff, J. & Weitz, J. S. (2013) Robust estimation of microbial diversity in theory and in practice. *ISME J.* **7**, 1092-1101.

6 Shade, A. (2017) Diversity is the question, not the answer. *ISME J.* **11**, 1-6.
