# Supplementary material for: Press Disturbance Alters Community Structure and Assembly Mechanisms of Bacterial Taxa and Functional Genes in Mesocosm-Scale Bioreactors
Source: mSystems. 2020 Aug 25;5(4):e00471-20. doi: 10.1128/mSystems.00471-20 (PMC7449608; doi:10.1128/mSystems.00471-20)
Supplement: TABLE S1 [file mSystems.00471-20-st001.pdf]

**Day**	**Phase**[†]	***n***	**F:M*** [mg-COD mg-TSS^{-1} d^{-1}]	**C:N** [mg-COD mg-TKN^{-1}]	**COD** [mg L^{-1}]	**TKN** [mg L^{-1}]	**TSS** [mg L^{-1}]	**SRT**[#] [d]
1-53	Acclimation	4	0.21 (0.08)	3.5 (0.7)	374 (106)	105 (27)	1934 (502)	11.6 (0.4)
54-127	Low organic loading	4	0.19 (0.05)	3.5 (0.3)	323 (24)	92 (3.6)	1727 (251)	7.9 (0.2)
54-113	High organic loading[‡]	3	0.36 (0.11)	6.3 (0.9)	629 (67)	100 (19)	1943 (476)	5.1 (0.9)
114-127	High-to-low organic loading[‡]	3	0.19 (0.06)	3.6 (0.3)	326 (19)	90 (2.2)	1774 (256)	7.7 (0.6)

[*] Average values, with standard deviation of the mean (s.d.m.) in parentheses.

[†] Each phase represents *n* replicates of independent 5-L reactors. Samples were generated 2-3 times per week.

[‡] These two phases involved the same reactors, where organic loading was changed from high to low on d114.

[#] The aerobic SRT, corresponding with the aerobic portion (61.8%) of each cycle.

F:M, food-to-biomass ratio

C:N, carbon-to-nitrogen ratio

COD, chemical oxygen demand

TKN, total Kjeldahl nitrogen

TSS, total suspended solids

SRT, solids residence time
