# Supplementary material for: Press Disturbance Alters Community Structure and Assembly Mechanisms of Bacterial Taxa and Functional Genes in Mesocosm-Scale Bioreactors
Source: mSystems. 2020 Aug 25;5(4):e00471-20. doi: 10.1128/mSystems.00471-20 (PMC7449608; doi:10.1128/mSystems.00471-20)
Supplement: TABLE S2 [file mSystems.00471-20-st002.pdf]

Sequencing method	Factor*	Nº of levels	*n*[‡]	df[§] res	PERMANOVA[†]		PERMDISP[†]	
					**F**	**P (MC)[¶]**	**F**	**P (perm)**
16S rRNA gene metabarcoding<br>ASV taxonomic level	t (d1-d47)	2	4	6	30.511	**0.0036**[‖]	180.44	0.0576
	OL (d56)	2	4, 3	5	1.6269	0.2942	0.2582	0.9006
	OL (d75)	2	4, 3	5	9.9127	**0.0086**	0.4442	0.5912
	OL (d96)	2	4, 3	5	8.9456	**0.0098**	2.7755	0.4470
	OL (d110)	2	4, 3	5	10.429	**0.0080**	1.5584	0.4715
	OL (d124)	2	4, 3	5	12.032	**0.0078**	0.5991	0.6363
Metagenomics shotgun sequencing<br>Genus taxonomic level	t (d1-d47)	2	4	6	193.11	**0.0036**	8.34	0.1948
	OL (d56)	2	4, 3	5	3.56	0.0576	0.0597	0.9375
	OL (d75)	2	4, 3	5	15.2	**0.0080**	1.5199	0.4715
	OL (d96)	2	4, 3	5	13.1	**0.0078**	0.8222	0.6532
	OL (d110)	2	4, 3	5	14.8	**0.0054**	0.2168	0.8984
	OL (d124)	2	4, 3	5	11.5	**0.0080**	0.7351	0.7343
Metagenomics shotgun sequencing<br>IP2G lowest gene level	t (d1-d47)	2	4	6	24.651	**0.0048**	29.441	0.0576
	OL (d56)	2	4, 3	5	1.8437	0.2292	0.0831	0.9006
	OL (d75)	2	4, 3	5	6.2232	**0.0180**	6.943	0.1097
	OL (d96)	2	4, 3	5	6.9173	**0.0174**	2.3795	0.4715
	OL (d110)	2	4, 3	5	7.14	**0.0168**	0.0006	0.9375
	OL (d124)	2	4, 3	5	6.3847	**0.0211**	0.8520	0.5912

* Factors (levels): t, time (d1 - d47) and OL, organic loading (low - high).
† Number of permutations used was 9,999
‡ Number of replicates per level
§ Degrees of freedom of the residual
¶ Approximate P-value from Monte Carlo permutations
‖ In bold, significant P-values after correction for multiple comparisons at a False Discovery Rate of 5%, using Benjamini-Hochberg's method.
