# Supplementary material for: Press Disturbance Alters Community Structure and Assembly Mechanisms of Bacterial Taxa and Functional Genes in Mesocosm-Scale Bioreactors
Source: mSystems. 2020 Aug 25;5(4):e00471-20. doi: 10.1128/mSystems.00471-20 (PMC7449608; doi:10.1128/mSystems.00471-20)
Supplement: TABLE S3 [file mSystems.00471-20-st003.pdf]

#	L	n	d	All (100% reads)						Common (90% acc. reads)					Rare (<10% acc. reads)				
				γ	α	β_{obs}	β_{exp}	DS (%)	β_{exp}: β_{obs}	γ	α	β_{obs}	β_{exp}	DS (%)	γ	α	β_{obs}	β_{exp}	DS (%)
**A**	A	4	1	448	211	0.529	0.075	85.8	0.14	222	123	0.447	0.113	74.7	274	88	0.680	0.149	78.1
			11	359	186	0.482	0.094	80.5	0.19	159	99	0.379	0.002	99.5	242	86	0.646	0.146	77.4
			25	227	126	0.447	0.084	81.2	0.19	92	60	0.348	0.000	100	159	64	0.601	0.106	82.4
			47	151	90	0.407	0.067	83.6	0.16	57	43	0.241	0.000	100	112	46	0.594	0.085	85.7
	L	4	56	152	86	0.436	0.058	86.8	0.13	62	42	0.323	0.000	100	109	43	0.606	0.088	85.5
			67	236	130	0.451	0.121	73.1	0.27	87	60	0.313	0.000	99.9	173	68	0.607	0.168	72.3
			75	204	122	0.402	0.091	77.3	0.23	83	58	0.301	0.000	100	143	63	0.558	0.122	78.1
			85	241	132	0.451	0.082	81.8	0.18	98	62	0.372	0.000	100	174	69	0.603	0.131	78.3
			96	164	94	0.427	0.054	87.3	0.13	68	45	0.335	0.000	100	121	48	0.605	0.082	86.5
			103	203	113	0.443	0.068	84.6	0.15	76	53	0.303	0.000	100	151	60	0.604	0.099	83.6
			110	183	81	0.560	0.036	93.6	0.06	77	40	0.481	0.000	100	125	40	0.682	0.056	91.7
			117	232	133	0.429	0.107	75.1	0.25	87	56	0.359	0.000	100	180	75	0.583	0.143	75.5
			124	210	121	0.423	0.079	81.4	0.19	84	54	0.363	0.000	100	164	66	0.596	0.115	80.8
	H	3	56	133	88	0.336	0.014	95.7	0.04	59	43	0.266	0.000	100	88	45	0.489	0.020	96.0
			67	205	136	0.337	0.048	85.8	0.14	99	72	0.276	0.000	100	135	63	0.533	0.066	87.6
			75	176	121	0.311	0.046	85.0	0.15	86	64	0.260	0.000	100	113	57	0.496	0.077	84.4
			85	185	124	0.328	0.051	84.5	0.16	86	62	0.279	0.000	100	121	62	0.490	0.088	82.1
			96	270	152	0.436	0.127	70.9	0.29	103	67	0.346	0.000	100	179	79	0.561	0.150	73.2
			103	179	115	0.359	0.032	91.0	0.09	83	57	0.309	0.000	100	117	57	0.513	0.058	88.7
			110	187	99	0.469	0.036	92.3	0.08	94	49	0.479	0.000	100	120	49	0.589	0.045	92.4
	H*	3	117	187	119	0.362	0.053	85.3	0.15	79	56	0.295	0.000	100	129	62	0.522	0.077	85.2
			124	181	116	0.357	0.036	89.8	0.10	83	56	0.325	0.000	100	127	60	0.530	0.080	84.9
**B**	A	4	1	946	852	0.100	0.031	68.8	0.31	188	173	0.082	0.015	82.1	785	679	0.135	0.038	72.1
			47	849	759	0.107	0.057	46.4	0.54	89	79	0.115	0.002	98.1	781	680	0.130	0.062	52.0
	L	4	56	831	744	0.105	0.049	53.0	0.47	101	92	0.089	0.004	95.5	747	652	0.127	0.055	57.0
			75	865	769	0.111	0.070	37.0	0.63	92	77	0.166	0.004	97.7	798	693	0.132	0.076	42.8
			96	915	815	0.110	0.077	29.8	0.70	82	72	0.122	0.002	98.7	856	743	0.132	0.082	37.8
			110	849	748	0.119	0.070	41.0	0.59	80	59	0.263	0.004	98.4	808	689	0.148	0.074	49.9
			124	870	764	0.122	0.087	28.9	0.71	70	49	0.304	0.004	98.7	838	715	0.147	0.090	38.6
	H	3	56	820	743	0.094	0.037	60.6	0.39	97	92	0.055	0.001	99.0	734	651	0.113	0.041	63.2
			75	822	748	0.090	0.030	66.7	0.33	115	103	0.104	0.001	98.6	731	645	0.118	0.034	71.3
			96	845	762	0.098	0.043	56.2	0.44	101	85	0.162	0.002	98.8	774	677	0.125	0.047	62.4
			110	890	783	0.120	0.071	41.3	0.59	86	68	0.205	0.001	99.5	798	685	0.142	0.063	55.3
	H*	3	124	890	783	0.120	0.071	41.3	0.59	79	62	0.215	0.001	99.6	846	721	0.148	0.074	49.8
**C**	A	4	1	3013	2634	0.126	0.157	24.4	1.24	1075	999	0.071	0.042	41.0	2093	1635	0.219	0.225	3.0
			47	2563	2332	0.090	0.122	34.7	1.35	1033	979	0.053	0.028	47.1	1631	1353	0.170	0.191	12.0
	L	4	56	2518	2346	0.069	0.102	48.6	1.49	1037	983	0.052	0.027	48.8	1587	1363	0.141	0.162	14.2
			75	2440	2245	0.080	0.105	31.5	1.31	1001	951	0.050	0.027	46.1	1536	1295	0.157	0.167	6.2
			96	2388	2212	0.074	0.098	33.8	1.34	1021	966	0.054	0.027	49.8	1477	1246	0.156	0.159	1.8
			110	2395	2205	0.080	0.104	30.5	1.31	1047	974	0.070	0.032	54.0	1495	1231	0.177	0.166	5.8
			124	2399	2215	0.077	0.101	31.2	1.31	1054	981	0.069	0.033	52.9	1490	1234	0.172	0.163	5.6
	H	3	56	2481	2351	0.053	0.083	57.5	1.57	1032	982	0.048	0.022	55.2	1546	1369	0.115	0.133	15.7
			75	2422	2254	0.070	0.096	38.0	1.38	981	925	0.057	0.022	61.9	1556	1329	0.146	0.149	2.1
			96	2344	2211	0.057	0.082	43.7	1.44	1010	958	0.052	0.020	61.3	1443	1253	0.132	0.133	1.0
			110	2343	2202	0.060	0.079	32.3	1.32	1030	970	0.059	0.023	60.9	1437	1233	0.142	0.130	8.9
	H*	3	124	2434	2255	0.074	0.094	27.8	1.28	1056	985	0.068	0.025	62.9	1529	1270	0.169	0.150	11.5

[#] Datasets: (**A**) 16 rRNA metabarcoding ASVs, (**B**) metagenomics genera, (**C**) metagenomics IP2G functional genes. [†] Phases: A, acclimation; L, low organic loading; H, high organic loading; H*, shift from high to low organic loading. [‡] Number of independent replicates. [§] Time (days). [¶] Null model parameters: $\boldsymbol{\gamma_{obs}}$, observed gamma diversity; $\overline{\boldsymbol{\alpha_{obs}}}$, mean observed alpha diversity; $\boldsymbol{\beta_{obs}}$, observed beta diversity; $\boldsymbol{\beta_{obs}}$, observed beta diversity; DS, deterministic strength; $\overline{\boldsymbol{\beta_{exp}}}$:$\boldsymbol{\beta_{obs}}$, expected (mean) to observed beta diversity ratio.
